# Immune Checkpoint Inhibitors as Monotherapy or Within a Combinatorial Strategy in Advanced Hepatocellular Carcinoma

**DOI:** 10.3390/ijms21176302

**Published:** 2020-08-31

**Authors:** Michela Guardascione, Giuseppe Toffoli

**Affiliations:** Experimental and Clinical Pharmacology Unit, Centro di Riferimento Oncologico di Aviano (CRO) IRCCS, 33081 Aviano, Italy; gtoffoli@cro.it

**Keywords:** hepatocellular carcinoma, liver tolerance, immune checkpoint inhibitors, combinatorial immunotherapy strategies

## Abstract

In advanced-stage hepatocellular carcinoma (HCC), systemic treatment represents the standard therapy. Target therapy has marked a new era based on a greater knowledge of molecular disease signaling. Nonetheless, survival outcomes and long-term response remain unsatisfactory, mostly because of the onset of primary or acquired resistance. More recently, results from clinical trials with immune targeting agents, such as the immune checkpoint inhibitors (ICIs), have shown a promising role for these drugs in the treatment of advanced HCC. In the context of an intrinsic tolerogenic liver environment, since HCC-induced immune tolerance, it is supported by multiple immunosuppressive mechanisms and several clinical trials are now underway to evaluate ICI-based combinations, including their associations with antiangiogenic agents or multikinase kinase inhibitors and multiple ICIs combinations. In this review, we will first discuss the basic principles of hepatic immunogenic tolerance and the evasive mechanism of antitumor immunity in HCC; furthermore we will elucidate the consistent biological rationale for immunotherapy in HCC even in the presence of an intrinsic tolerogenic environment. Subsequently, we will critically report and discuss current literature on ICIs in the treatment of advanced HCC, including a focus on the currently explored combinatorial strategies and their rationales. Finally, we will consider both challenges and future directions in this field.

## 1. Introduction

Liver cancer incidence consists of about 850,000 new diagnoses per year; nearly 90% of primary liver tumors are HCC [1] representing one of the leading causes of cancer-related death. The dominant risk factors for HCC vary worldwide, being hepatitis B virus infection and aflatoxin B1 exposure for most countries in Asia and Africa; in contrast, hepatitis C virus infection, alcoholism, and metabolic syndrome represent major risk factors in other areas of the world [2]. The Barcelona Clinic Liver Cancer (BCLC) staging criteria represent the main clinical classification that stratifies patients according to prognosis, in order to better formulate treatment strategies. Early-stage cancers are potentially suitable for therapies with curative intent such as surgical resection, liver transplantation, or local ablation. Trans-arterial chemoembolization (TACE) and systemic therapy represent the only therapeutic options for intermediate/advanced-stages disease HCC [1]. Trans-arterial radioembolization (TARE) is an alternative embolization approach with a favorable safety and efficacy profile, however well-designed, properly powered randomized clinical trials are still needed [1].

In advanced-stage or intermediate when chemoembolization is no longer indicated, systemic treatment represents the standard therapy for HCC. Conventional chemotherapy has shown unsatisfactory results, while target therapy has marked a new era for the treatment of patients with HCC based on a greater knowledge of molecular signaling of HCC [3,4]. At present, four orally administered small-molecule multikinase kinase inhibitors (MKIs), namely sorafenib, lenvatinib, regorafenib, and cabozantinib have been approved in Europe for advanced HCC indication. More specifically, sorafenib and lenvatinib have been approved as first-line therapy and regorafenib and cabozantinib in patients who have progressed to sorafenib. Furthermore, on 27 June 2019, based on the results from the phase III REACH-2 trial [5], the human monoclonal antibody (mAb) against *Vascular Endothelial Growth Factor Receptor 2* (VEGFR2) ramucirumab was approved for selected patients with HCC with serum alpha fetoprotein (AFP) of ≥400 ng/mL and who have been previously treated with sorafenib.

Sorafenib is a small molecule that inhibits a number of serine/threonine and tyrosine kinases such as VEGFR1–3, *platelet-derived growth factor receptor* (PDGFR), *fibroblast growth factor receptor 1* (FGFR1), *KIT proto-oncogene receptor tyrosine kinase* (KIT), *RET proto-oncogene* (RET), *FMS-related tyrosine kinase 3* (FLT3), and downstream *RAF proto-oncogene* signaling players (e.g., RAF-1, BRAF). Thus, it affects multiple oncogenic pathways, such as angiogenesis and tumor proliferation [6]. Phase III SHARP trial evaluating sorafenib in previously untreated patients with advanced HCC reported a significant improvement in median overall survival (OS) compared to placebo (10.7 vs. 7.9 months) [7]. Although survival improvement has been achieved with this targeted agent, only a limited number of patients have experienced a long-term benefit. Indeed, the onset of drug resistance and/or the occurrence of significant toxicities have restricted by the advantages of sorafenib. Regorafenib, similarly to sorafenib, inhibits multiple kinases including VEGFR1, –2, –3, KIT, RET, BRAF, PDGFR, FGFR. Phase III RESORCE trial reported an OS benefit for regorafenib compared to placebo (10.6 vs. 7.8 months) in patients who tolerated but progressed on sorafenib [8]. Lenvatinib is another MKI that inhibits VEGFR1–3, FGFR 1–4, PDGFRα, RET, and KIT. Phase III REFLECT trial comparing lenvatinib to sorafenib in the first-line setting reached its OS non-inferiority primary endpoint, also showing a statistically significant improvement for secondary end-point progression-free survival (PFS); furthermore, fewer dermatological adverse events with more hypertension occurred with lenvatinib [9]. Finally, cabozantinib is able to block PDGFR, HGFR, VEGFR2, AXL, RET, KIT, and FLT3. This drug was associated with longer OS than placebo in a phase III trial involving patients already treated for advanced disease [10]. Another MKI that should be mentioned for completeness is tivantinib, a selective inhibitor of the proto-oncogene MET. A phase II randomized trial with tivantinib as a second-line treatment for patients with HCC showed improved PFS for tivantinib compared with placebo in a subcohort of patients with high MET expression tumors [11]. On 13 November 2013, based on these results, orphan designation (EU/3/13/1202) was granted for the use of tivantinib in patients who were resistant to sorafenib. However, a subsequent phase III trial evaluating the use of tivantinib for second-line treatment of MET-high expressing advanced HCC showed no OS improvement for tivantinib compared with placebo in patients previously treated with sorafenib [12].

Over the last few years, a drug class called immune checkpoint inhibitors (ICIs), has emerged to play a promising role in the treatment of several cancers, including HCC. The immune response is, in fact, a key regulator of tumor biology with the ability to both support and inhibit tumor development, proliferation, metastasization. T cells are able to recognize malignant cells and address them to be destructed. While the immune response to specific antigen is recognized by *major histocompatibility complex* (MHC) receptors, the intensity of the response is regulated by costimulatory and coinhibitory molecules. Immune checkpoints are negative regulators of T cells that are physiologically expressed for the maintenance of self-tolerance, to guarantee that the immune response is not permanently activated. Among them *programmed death-1* (PD-1), *cytotoxic T-lymphocyte antigen 4* (CTLA-4), *T-cell immunoglobulin and mucin domain-containing protein 3* (TIM-3) and *Lymphocyte-activation gene 3* (LAG-3) have been studied in chronic hepatitis and HCC. To date, two immune checkpoint pathways have been best characterized. The first one is the B7-1 or B7-2/CTLA-4 pathway. B7-1 and B7-2 are expressed on antigen (Ag) presenting cells (APCs) and can interact with CTLA-4 expressed by T cells. The second one is the PD-1/programmed death-ligand 1 (PD-L1, also referred to as B7-H1) or *programmed death-ligand 2* (PD-L2, also referred to as B7-DC) pathway. PD-1 is expressed on activated T cells, B cells, monocytes, dendritic cells (DCs), and, at low levels, on natural killer T cells (NKT). Signaling induced by PD-1 and CTLA-4 are not redundant: PD-1 plays its main roles in peripheral tissues by modulating inflammatory responses while CTLA-4 regulates T-cell priming in lymphoid organs. Furthermore, in contrast to CTLA-4, PD-1 is able to inhibit TCR- and CD28-mediated activation [13]. Two known PD-1 ligands, belonging to the B7 family, are PD-L1 and PD-L2. These two ligands differ from each other mainly because of their expression pattern. In fact, PD-L1 is expressed by activated T cells, B cells, monocytes, DCs, endothelial cells, whereas PD-L2 expression is largely restricted to activated macrophages and DCs [13]. However, tumor cells have the capability to escape from immunosurveillance; ICIs are able to inhibit negative regulators of T cells, thus enhancing T cell-mediated antitumor immunity. To date, several ICI targeting PD-1/PD-L1 and CTLA-4 have showed promising results in clinical trials in terms of activity and manageable toxicity, thus receiving approval by the US Food and Drug Administration (FDA). In particular, the ICIs approved by the FDA for various oncology indications are seven, including metastatic melanoma, non-small-cell lung cancer, urothelial carcinoma, and renal cell carcinoma: ipilimumab and tremelimumab which are CTLA-4 inhibitors; the anti-PD-1 agents nivolumab and pembrolizumab; and atezolizumab, avelumab, and durvalumab that act as PD-L1 inhibitors. Numerous clinical trials are underway to study the safety and efficacy of these agents in other solid and hematological malignancies, including HCC. Furthermore, combinatorial therapeutic strategies involving ICIs are also showing interesting data in clinical studies.

In this review, we will first discuss the basic principles of physiological hepatic immunogenic tolerance and the pathological evasive mechanism of antitumor immunity in HCC; second we will elucidate the biological rationale for immunotherapy in HCC. Furthermore, we critically report and discuss current literature on ICIs in the treatment of advanced HCC, with a specific focus on the currently explored combinatorial strategies and their rationales. Finally, we will consider both challenges and future directions in this field.

## 2. Basic Principles of Hepatic Immune Response

### 2.1. Liver Immune Tolerance

**Figure 1:**
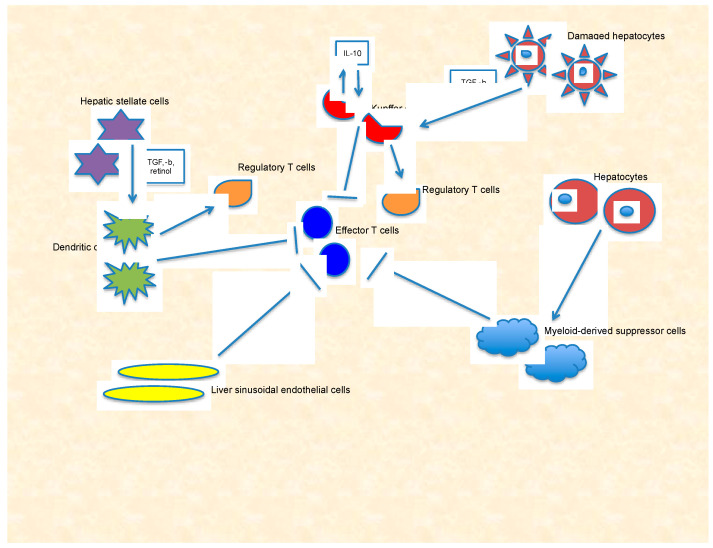
Immune tolerance in the liver is maintained by multiple subsets of cells: Kupffer cells, liver sinusoidal endothelial cells, dendritic cells, hepatic stellate cells, and hepatocytes. Damaged hepatocytes release *transforming growth factor* (TGF)-b stimulating KCs to secrete *interleukin* (IL)-10 that acts in an autocrine manner to induce immunosuppression through several mechanisms involving both effector T cells and regulatory T cells. Healthy hepatocytes promote the function of myeloid-derived suppressor cells whereas hepatic stellate cells secrete retinol and TGF-b, promoting regulatory T cells’ activation in the presence of dendritic cells. Finally, liver sinusoidal endothelial cells are a particular type of resident antigen presenting cells with a reduced capability to activate effector T cells.

Since physiologically involved in functions such as metabolism and excretion of toxics, filtration of environmental or bacterial microorganisms from the gastrointestinal tract, the liver is subjected to an enormous antigen exposure. In order to avoid an autoimmune damage, liver evolution resulted in developing intrinsic immune tolerance, both in the innate and in the adaptive immune response [14]. Immune response in the liver is maintained by multiple subsets of cells: resident macrophages (Kupffer cells, KCs), liver sinusoidal endothelial cells (LSECs), DCs, hepatic stellate cells (HSCs), and hepatocytes (Figure 1). Under physiological conditions, in order to maintain tolerogenicity, KCs, belonging to the innate immune system, eliminate high-affinity antigen-specific CD8^+^ T cells entered into the liver; they produce *interleukin* (IL)-10 and *transforming growth factor* (TGF)-b that play an inhibitory role on immune response [15,16,17]. LSECs are a resident APCs population in the liver; they transport exogenous antigens into liver parenchyma and present both MHC class I and II (MHC-I and -II) molecules. It has been demonstrated that endotoxins affect Ag processing and expression of the accessory molecules in these cells, thus limiting their ability to activate CD4^+^ T cells and resulting in a reduced function of adaptive immune system surveillance in the liver [16]. In the context of immune checkpoint pathways, with respect to the immunosuppressive role of CTLA-4 in the liver, CTLA-4 expression by Foxp3^+^CD25^+^CD4^+^ T regulatory cells (Tregs) has been linked to the induction of host immune tolerance after liver transplantation [18] and, thus represents a potential organ-specific mechanism to regulate immune activation. In addition, the immunosuppressive role of PD-L1 in the liver contributes to the mechanisms of immune tolerance. In fact, PD-L1 is expressed on hepatocytes [19], HSCs [20], LSECs [21], Kupffer cells [22], and mediate T cell apoptosis [19,20] or T-cell dysfunction [21].

From a certain point of view, all these tolerogenic mechanisms that occur in the liver can be protective with respect to harmless antigens or in order to prevent an autoimmune organ damage. However, the same processes can result in a detrimental effect in case of immune tolerance to tumor-associated antigens (TAAs) in HCC. Moreover, in the context of chronic hepatitis, such as viral and autoimmune hepatitis, the chronic inflammatory state inhibits Ag-specific immune surveillance, by inducing changes in the expression of immune checkpoints [23,24], alterations of DCs [25,26], increase of Tregs [27,28], and release of cytokines with immune suppressive functions such as IL-10 and TGF-b [23,28]. More specifically, upregulation of PD-1 expression on intrahepatic lymphocytes and its ligands PD-L1 and PD-L2 on Kupffer cells, LSECs, and leukocytes have been positively correlated with the degree of liver inflammation [22]. In addition to the PD-1 upregulation, other immune inhibitory checkpoints such as CTLA-4 [21] and TIM-3 [29] have been linked to reduced T cell effector function in chronic viral hepatitis. Collectively, these findings suggest that an immunosuppression occurs in the hepatic chronic inflammatory state. It appears reasonable to hypothesize that this condition may allow HCC onset and progression; therefore, immune targeting treatments might allow the restoration of T-cell functions in chronically inflamed livers and thus prevent HCC development.

### 2.2. HCC Immune Tolerance

**Figure 2:**
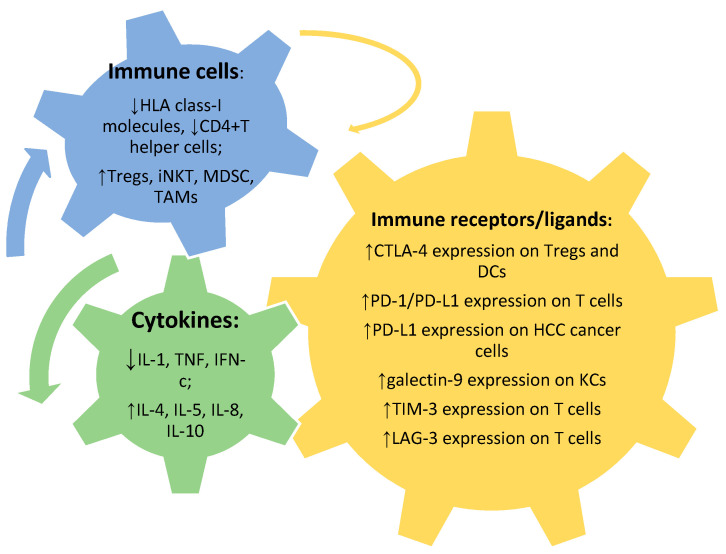
In addition to the tolerogenic hepatic environment, multiple players can contribute to immune evasion in HCC: immune cells, cytokines, immune receptors or ligands. Abbreviations: *human leukocyte antigen* (HLA); regulatory T cells (Tregs); invariant natural killer T cells (iNKT); myeloid-derived suppressor cells (MDSC); tumor-associated macrophages (TAMs); *cytotoxic T-lymphocyte antigen 4* (CTLA-4); dendritic cells (DCs); *programmed death-1* (PD-1); *programmed death-ligand 1* (PD-L1); *T-cell immunoglobulin and mucin domain-containing protein 3* (TIM-3); *Lymphocyte-activation gene 3* (LAG-3); *interleukin* (IL); *tumor necrosis factor* (TNF); *interferon* (IFN).

In addition to the tolerogenic hepatic environment, multiple mechanisms can contribute to immune evasion in HCC, involving several players including immune cells, cytokines, immune receptors, or ligands (Figure 2), as well as different mechanisms. In the context of immune cells, a decreased expression of *human leukocyte antigen* (HLA) class-I molecules and consequently failure of Ag presentation by APCs [30], an impaired tumor antigen processing [31], an increase in Tregs [32], invariant natural killer T cells (iNKT) [33], CD14^+^HLA-DR^−/low^, monocytic-like myeloid-derived suppressor cells (MDSC) [34], tumor-associated macrophages (TAMs) [35], as well as a reduced CD4^+^ T helper cells have been reported [36]. Furthermore, the number of NK cell infiltration was found to inversely correlate with HCC progression [37]. It has been reported that, within the NK cells, the subpopulation of CD56^dim^CD16^+^ was particularly low in HCC [38]. As far as cytokine level is concerned, HCC microenvironment has been shown to present a unique innate immunity signature which includes an increase in immuno-suppressive cytokines (IL-4, IL-5, IL-8, and IL-10) associated with a reduction of immune-stimulating cytokines (IL-1, tumor necrosis factor (TNF), and *interferon* gamma (IFN-c) [39]. With respect to receptors/ligands, tumor-induced immune tolerance is mediated by changes in the expression of several inhibitory checkpoints including CTLA-4 [40,41,42], PD-1, and its ligand (PD-L1) [43], LAG-3 [44], TIM-3 and its ligand (galectin-9) [45], and *adenosine A2a receptor* (A2aR) [46]. In details, high CTLA-4 expression on Tregs in peripheral blood has been reported in patients with HCC [41]. In addition, CTLA-4 expression on CD14^+^DCs was found to be associated with IL-10 and indoleamine-2,3-dioxygenase (IDO)-mediated inhibition of T-cell proliferation and induction of T-cell apoptosis [40]. In the context of PD-1/PD-L1 pathway, a high expression levels of PD-1/PD-L1 as well as of markers of inflammatory response on HCC immune cells infiltrates has been demonstrated [43]; furthermore, an increase in tumor infiltrating and circulating PD-1^+^CD8^+^ T cells has been associated with disease progression after curative hepatic resection [47]. In addition to the upregulation of PD-1 on T cells, its ligand, PD-L1, was found to be highly expressed on HCC cancer cells [43,48,49]. Finally, in HBV-related HCC, KCs can express galectin-9, which interacts with TIM-3 on T cells and inhibits immune response in HCC [50].

### 2.3. Rationale for Immunotherapy in HCC

Several evidences assume that HCCs are potentially immunogenic. It has been demonstrated that the HCC tumor microenvironment (TME) contains tumor infiltrating lymphocytes (TILs). In particular, CD8^+^ T-cell tumor infiltrate was found to correlate with low recurrence rate after HCC surgery [51]. T-cell infiltration has also been reported after percutaneous ethanol injection or radiofrequency ablation [52]. Moreover Flecken et al. showed that CD8^+^ T cells in HCC could be found in more than 50% of patients and that infiltration burden correlated with patient outcome [53]. Furthermore, other reports mentioned the presence in HCC lesions of TILs expressing PD-1 and their possible correlation with positive outcome [54]. In another study, CD8^+^ T cells in HCC were distinct in subgroups with a range of PD-1 expression levels: PD-1-high, PD-1-intermediate, and PD-1-negative. The presence of CD8^+^ T cells with high PD-1 expression (PD-1^high^ CD8^+^) was reported to correlate with the aggressiveness of HCC and to potentially predict for anti-PD-1 therapeutic response [55].

## 3. ICI Monotherapy

The main clinical trials with ICIs in monotherapy for the treatment of HCC are summarized in Table 1 and Table 2.

### 3.1. Anti-PD-1

#### 3.1.1. Nivolumab

Nivolumab is a fully human immunoglobulin (Ig) G4 (IgG4) mAb. Its binding to PD-1 receptor on T-cells inhibits the interaction with PD-L1 and PD-L2 on tumor cells and thus leads to restore the antitumor activity of T-cells [56]. A non-comparative phase I/II study (CheckMate 040) with nivolumab was conducted in patients with histologically confirmed advanced HCC; previous sorafenib treatment was allowed. The trial included 262 patients, 48 of them in the dose-escalation phase and 214 of them in the dose- expansion phase. In the dose-expansion phase, overall response rate (ORR) and disease control rate (DCR) were 20% and 64% respectively, and progression free survival (PFS) was 4.1 months, with a manageable safety profile [57]. On September 2017, based on the results of this study, the FDA granted accelerated approval of nivolumab as a second-line treatment for unresectable HCC after sorafenib failure, marking the start of ICI approval for HCC indication in the United States. Topline results from a subsequent randomized phase III trial (CheckMate 459) with first-line nivolumab compared with sorafenib in 743 patients with histologically confirmed advanced HCC that was ineligible to or progressing after surgical and/or locoregional therapies revealed that the study did not meet its primary endpoint of improved OS (HR 0.84, *p*  =  0.0419). In this study clinical benefit was observed across predefined subgroups, including hepatitis infection status, presence of vascular invasion and/or extrahepatic spread, and region (Asia vs. non-Asia). ORR was 15% for nivolumab (14 patients with Complete Response-CR) and 7% for sorafenib (5 patients with CR). Grade 3/4 treatment-related adverse events were reported in 22% of patients in the nivolumab arm and 49% in sorafenib group [58]. Regardless, the trial revealed a clear trend toward an OS improvement with nivolumab versus sorafenib, and thus exploration of nivolumab in HCC will continue. The drug is being evaluated in other studies, such as in the adjuvant setting in patients with HCC at high risk of recurrence after curative hepatic resection or ablation (CheckMate-9DX; NCT03383458).

#### 3.1.2. Pembrolizumab

Pembrolizumab is another anti-PD-1 IgG4 mAb but in contrast to nivolumab it is a humanized antibody. In 2016, Truong et al. published the first case report of response to compassionate pembrolizumab in advanced HCC after sorafenib failure [59]. Since then, several studies have continued investigating pembrolizumab in patients with advanced HCC who progressed on or were intolerant to first-line systemic therapies. In particular, a non-randomized phase II trial (KEYNOTE-224) showed 1%, 16%, and 44% of complete response, partial response, and stable disease rates respectively. Therefore an ORR of 17% and a DCR of more than 60% were achieved in this trial, with a median OS of 12.9 months and a favorable safety profile [60]. Considering the results of this study, on November 2018 the FDA granted accelerated approval of pembrolizumab as a second-line treatment after sorafenib, starting also a priority review program for second-line pembrolizumab in HCC. Therefore a subsequent randomized, placebo-controlled phase III trial (KEYNOTE-240) was conducted. However, the trial failed prematurely since it did not reach the pre-specified statistical criteria, although an improved PFS compared with placebo was reported and ORR and safety profile data were consistent with those of KEYNOTE-224 [61], supporting further research with pembrolizumab in patients with HCC. In particular, two further phase III trials are currently ongoing: the first one (KEYNOTE-394) is evaluating pembrolizumab versus placebo in Asian patients with pre-treated advanced HCC, while the other study (KEYNOTE-937) is investigating pembrolizumab versus placebo as an adjuvant therapy in patients with HCC after curative treatment.

#### 3.1.3. Tislelizumab

The investigational IgG4 anti-PD-1 mAb tislelizumab (BGB-A317), was properly designed to minimally bind to FcR on macrophages; its peculiar pharmacodynamic features make the drug able to overcome antibody-dependent phagocytosis, which is a potential mechanism of anti-PD-1 therapy resistance [62]. Report of the HCC cohort of a phase I trial with tislelizumab showed an ORR of 12.2% and a DCR of 51.0%; the most common treatment-emergent AEs were decreased appetite, rash, decreased weight, and cough [63]. This preliminary safety profile and antitumor activity supported continuing development of tislelizumab in HCC and thus a phase III trial (RATIONALE 301, NCT03412773) of tislelizumab versus sorafenib as a first-line treatment is currently underway.

### 3.2. Anti-PD-L1

The human IgG1 mAb durvalumab was tested within a phase I/II trial in patients with HCC already treated with sorafenib, achieving an ORR of 10.3% [64]. Avelumab, another human IgG1 mAb, is currently being tested in an ongoing phase II study in patients with advanced HCC after prior sorafenib treatment (NCT03389126). Finally, atezolizumab is an anti-PD-L1 humanized IgG mAb. The phase Ib study GO30140 evaluating atezolizumab monotherapy compared to combination of atezolizumab and the anti-VEGF bevacizumab in patients with advanced HCC, showed a median PFS of 3.4 months in the monotherapy arm, compared to 5.6 months in the combination arm (HR 0.55, *p* = 0.018) [65].

### 3.3. Others

In the context of anti-CTLA-4, the fully human mAb tremelimumab has been tested in HCC. In particular, a small phase II study evaluated tremelimumab in patients with advanced HCC and chronic HCV infection. Results from this study showed that tremelimumab was a safe treatment, with a DCR of 76.4% and a median PFS of 6.48 months; in addition a significant decline in viral load was observed [66]. These findings suggest that tremelimumab could be particularly promising for hepatitis C-related advanced HCC. Moreover, the immune checkpoints TIM-3 and LAG-3 may represent potential targets for HCC immunotherapy. Indeed Yan et al. showed that TIM-3 was highly expressed by monocytes and macrophage in patients with HCC and TIM-3 knockdown in macrophages inhibited tumor growth both in vitro and in vivo [67]. As for LAG-3, its aberrant expression has been found in a broad spectrum of human tumors including HCC [68]. Therefore, targeting TIM-3 and LAG-3 might be promising approaches in the treatment of HCC.

## 4. ICI-Based Combinatorial Therapies

Since HCC-mediated immune tolerance, in the context of an intrinsic tolerogenic liver environment, is supported by multiple immunosuppressive mechanisms, it is reasonable to hypothesize that dual or triple combinations of immunotherapeutic could represent the most promising strategies for clinical development in advanced HCC. Indeed, several clinical trials are now underway to evaluate ICI-based combinations as a strategy to overcome primary and acquired resistance, and consequently improve patient outcome. In particular, randomized phase III trials are ongoing—or just completed—with various ICI-based combinatorial strategies, including associations between different ICIs such as PD-L1 plus CTLA-4 inhibition (HIMALAYA, NCT03298451) or combinations with antiangiogenic therapies utilizing both mAbs such as bevacizumab (IMbrave 150, NCT03434379) and MKIs such as lenvatinib (LEAP-002, NCT03713593) or cabozantinib (COSMIC-312, NCT03755791). Theoretically, sensitivity to ICIs could be enhanced by multiple strategies such as (i) priming adaptive responses through therapies that release tumor antigens (radiotherapy or chemotherapy) or cancer vaccines, (ii) increasing antigen presentation by intratumoral delivery of oncolytic virus or RNA adjuvants, (iii) with agents that inhibit VEGF and TGF-β, which are able to enhance dendritic cell function and decrease Tregs in the tumor microenvironment, or (iv) through agonistic antibodies that target immunostimulatory molecules such as CD40 or CD137. In this section we will mainly focus on ICI-based combinatorial strategies that are currently under clinical evaluation.

### 4.1. ICIs Combined with Antiangiogenic Drugs

VEGF overexpression has been associated with immunosuppressive effects in tumors via multiple direct and indirect mechanisms as shown both in preclinical and clinical studies. Data from studies of peripheral blood of patients who underwent bevacizumab treatment showed that anti-VEGF treatment increased the number of DCs [69], underlying a VEGF capability to inhibit maturation and proliferation of DCs. Moreover, VEGF overexpression can promote accumulation of immunosuppressive cells and molecules. In particular, increased intratumoral MDSCs infiltration was found to correlate with circulating VEGF levels in several tumor types including HCC [70]; in addition preclinical studies demonstrated that sunitinib [71] and sorafenib [72] could decrease MDSC levels in spleen, bone marrow and tumor. Sorafenib treatment has been reported to decrease also intratumoral Treg density and inhibit their function in mouse models of liver cancer [73]. Clinical data support the decrease in Tregs by anti-VEGF therapy in the treatment of colorectal carcinoma [74]. Furthermore, VEGF is able to down-regulate T-cell differentiation and its cytotoxic function [75] and thus T-cell infiltration and activity may be promoted by anti-VEGF therapy. In addition, considering that tumor vessels are a major port of entry for T-cells into tumors, although usually altered in their structure and function, they may result in inhibition of T-cell infiltration into tumor [76] and also in this case an anti-VEGF therapy could potentially revert this phenomenon. Combining anti-VEGF and immunotherapeutic drugs has shown promising synergy in some animal models. In particular, in a mouse model with subcutaneous implanted tumors the combination of anti-VEGFR2 and anti-PD-1 antibodies could promote IFN-γ, TNF-α and granzyme B production, suggesting the enhancement of immune response [77]. Furthermore, the CXCR4 inhibitor AMD3100 in combination with sorafenib was reported to inhibit tumor growth in another mouse model [78]. A subsequent analysis conducted with the same model, showed that immune PD-L1 expression in the tumor was increased after sorafenib treatment, suggesting a promising role for a triple combination therapy with AMD3100, sorafenib and anti-PD-1 antibody [79]. In the following paragraphs, we will discuss clinical evidences on the synergistic activity of major anti-VEGF drugs and ICIs.

#### 4.1.1. Bevacizumab

Bevacizumab is an anti-VEGF mAB that prevents VEGF from interacting with VEGF receptors on the surface of endothelial cells, thus suppressing tumor angiogenesis. To date, bevacizumab is not approved for HCC indication. Indeed a phase II trial with bevacizumab single agent in advanced HCC, although showing significant clinical activity, reported grade 3-4 astenia, hemorrhage, and aminotransferase elevation in 12%, 7%, and 7% of patients respectively [80], and thus no phase III trial of bevacizumab for HCC has been conducted so far. Further phase II studies of bevacizumab in advanced HCC included a trial evaluating the combination of gemcitabine, oxaliplatin, and bevacizumab, achieving a median OS of 9.6 months and a median and PFS of 5.3 months [81], and another study of bevacizumab plus erlotinib which did not show differences in efficacy compared to single agent sorafenib, although the safety profile tended to favor the combination [82]. With regard to combination with ICIs, phase Ib study of bevacizumab plus atezolizumab showed promising ORR [83]. Recent findings from the primary analysis of data from the phase III IMbrave 150 trial (NCT03434379) evaluating atezolizumab plus bevacizumab versus single agent sorafenib in patients with HCC who had not previously received systemic treatments, have reported a significant OS and PFS improvement in favor of the therapeutic association. Following 2:1 randomization, 336 patients were treated with atezoliumab plus bevacizumab and 165 received sorafenib. OS at 12 months was 67.2% (95% CI, 61.3 to 73.1) with atezolizumab–bevacizumab and 54.6% (95% CI, 45.2 to 64.0) with sorafenib. Median PFS was 6.8 months (95% CI, 5.7 to 8.3) and 4.3 months (95% CI, 4.0 to 5.6) in the respective groups (HR 0.59; 95% CI, 0.47–0.76; *p* < 0.0001). The ORR with the combination compared to sorafenib was 27% versus 12% (*p* < 0.0001). The safety of the combination was consistent with the known safety profile of each agent, and no new safety signals were identified [84]. Based on the findings from the IMbrave150 trial, the combination of atezolizumab and bevacizumab may be practice changing in the first-line setting for HCC. Within the context of combining bevacizumab and ICIs, further phase III studies are currently underway. In particular, IMbrave 050 trial (NCT04102098) is comparing the same combination of that in IMbrave 150 trial (atezolizumab+ bevacizumab) with active surveillance in patients with HCC at high risk of recurrence after curative treatment, while EMERALD-2 trial (NCT03847428) is evaluating the combination durvalumab + bevacizumab versus durvalumab alone in the same adjuvant setting. Finally, ORIENT-32 trial (NCT03794440) is currently exploring the association of IBI305 (bevacizumab biosimilar) with the fully human anti-PD-1 IgG4 mAb sintilimab compared to sorafenib monotherapy as first-line treatment for HCC.

#### 4.1.2. MKIs

A phase II trial with sorafenib and nivolumab as first-line therapy in HCC is currently ongoing (NCT03439891). In the context of lenvatinib, based on the REFLECT study results, it represents a first-line therapeutic option in patients with advanced HCC; combination of lenvatinib with ICIs is still under evaluation. Findings from phase Ib KEYNOTE-524 trial with lenvatinib plus pembrolizumab in unresectable HCC showed a good safety profile and an encouraging anti-tumor activity in unresectable HCC [85]. Based on the results of this phase Ib trial, on July 2019 the FDA granted breakthrough therapy designation for pembrolizumab in combination with lenvatinib in the first-line treatment for HCC. Consequently, phase III LEAP-002 trial evaluating lenvatinib + pembrolizumab vs. lenvatinib + placebo as a first-line therapy for advanced HCC is underway (NCT03713593). In addition to sorafenib and lenvatinib, further MKIs are being evaluating in HCC. In particular, a phase Ib trial was conducted with apatinib plus the anti-PD-1 antibody SHR-1210, showing promising ORR [86]. Moreover, a phase II trial in Chinese patients (NCT03092895) and a phase III trial (NCT03764293), both evaluating the same combination of apatinib and SHR-1210, are currently ongoing. Finally, a phase III trial exploring cabozantinib plus atezolizumab (NCT03755791/COSMIC-312) is currently under investigation.

### 4.2. ICIs Combined with Chemotherapy

It has been demonstrated that chemotherapeutic agents are able to promote anti-tumor immunity by induction of the immunogenic cell death (ICD). This phenomenon is characterized by the release of danger signals from tumor cells, which can polarize DCs toward a pro-inflammatory phenotype and thus drive a T helper 1 (Th1) response [87]. In addition to inducing immunogenic cell death, some chemotherapeutic drugs can downregulate Tregs and MDSCs, further promoting anti-tumor immune response. In particular, pre-clinical evidences reported that low-dose cyclophosphamide or gemcitabine could selectively deplete Tregs while 5-fluorouracil showed a pronounced effect on MDSC depletion [88]. In patient with advanced HCC, treatment with low-dose cyclophosphamide was found to impair regulatory Tregs [89]. Although HCC has been long considered highly refractory to conventional systemic chemotherapy, these and further evidences have provided a rationale for investigating the combination of chemotherapy with ICIs in cancer, including HCC. Indeed, a phase II trial of SHR-1210 combined with FOLFOX4 or GEMOX was conducted in Chinese patients with HCC, reporting an ORR of 26.5%, a DCR of 79.4% and a median PFS of 5.5 months (NCT03092895). A subsequent phase III trial evaluating SHR-1210 + FOLFOX4 as first-line therapy in patients with advanced HCC is currently underway (NCT03605706).

### 4.3. Dual Immune Checkpoint Blockade

Combinations of anti-PD-1/PD-L1 and anti-CTLA-4 mAb have been investigated in various tumor types, including HCC. Phase I/II CheckMate-040 study of nivolumab plus ipilimumab in HCC demonstrated that the combination had an acceptable safety profile and achieved clinically meaningful responses, with an ORR of 31%, a DCR of 54%, and a median OS of 22.8 months that is the longest duration of OS in the second-line setting for advanced-stage HCC tested in clinical trials so far [90]. In more details, CheckMate-040 trial had a first part in which nivolumab dose escalation phase was designed to establish the safety of the drug at different dose levels in three cohorts (uninfected subjects, hepatitis C virus-infected subjects, and hepatitis B virus-infected subjects). The second part of the study was the expansion phase designed to generate additional clinical data at specified doses for each of the three cohorts. Subsequently, further cohorts have been added to the study, in order to generate data on the safety and the efficacy of the following combinations in the treatment of advanced HCC: nivolumab plus sorafenib, nivolumab plus cabozantinib, nivolumab plus ipilimumab, and nivolumab in combination with both cabozantinib and ipilimumab. Based on data from this study, on November 2019 the FDA granted breakthrough therapy designation for nivolumab in combination with ipilimumab in patients with advanced HCC who had previously been treated with sorafenib. Currently, another trial (NCT04039607) investigating the combination nivolumab + ipilimumab versus standard of care sorafenib or lenvatinib in patients with HCC who have not previously received systemic treatments is ongoing. Furthermore, a phase I/II trial is underway to evaluate the combination of anti-OX40 mAb, a drug targeting the immune costimulatory molecule CD40, with nivolumab and ipilimumab in patients with HCC (NCT03241173). With respect to other combinations, durvalumab + tremelimumab has been evaluated in a phase I/II study in advanced HCC, achieving an ORR of 17.5% [91]. The combination was also well tolerated and thus a randomized phase III trial (NCT03298451) is currently investigating durvalumab monotherapy and durvalumab + tremelimumab versus sorafenib as a first-line treatment in unresectable HCC. In January 2020, the combination durvalumab plus tremelimumab has been granted orphan drug designation for HCC by FDA. Moreover, two phase I trials are currently evaluating the dual immune checkpoint blockade of LAG-3 and PD-1 (NCT03005782) and TIM-3 and PD-L1 (NCT03099109) in patients with HCC.

### 4.4. ICIs Combined with Locoregional Therapies

Locoregional therapies for HCC such as local ablation—by means of alcohol, radiofrequency (RFA), microwave, or cryoablation—TACE, TARE or less commonly radiotherapy, could promote anti-tumor immunity via the release of tumor-specific antigens from killing of tumor cells [92,93]. A number of preclinical proof-of-concept studies [94,95] suggested that combined use of locoregional therapies could improve the effectiveness of immunotherapeutic agents. With regards to translational studies, the number of AFP-specific T cells was observed to increase after TACE [92]; furthermore, immune-mediated abscopal effects of radiation have been reported in patients with HCC treated with external beam radiation [96] and TARE [97]. On a clinical level, a phase I/II study of RFA+ tremelimumab, conducted in 32 patients with advanced HCC, reported a DCR of 89%, with a median time to progression (TTP) and OS of 7.4 and 12.3 months respectively. Interestingly, 12 of 14 patients with quantifiable HCV experienced a marked reduction in viral load and had a clinical benefit from the therapy in terms of disease control; in contrast, two patients who had no viral reduction derived no benefit from the treatment, suggesting that antiviral immune responses may represent a surrogate for disease control. Moreover, in the same study the increase of intratumoral CD8+ T cells was found to be associated with a clinical benefit [98]. A phase III trial evaluating TACE in combination with durvalumab and bevacizumab in HCC is currently ongoing (NCT03778957). Furthermore, phase II trials investigating radiotherapy combined with ICIs in HCC are now underway, in particular stereotactic body radiation therapy (SBRT) with pembrolizumab (NCT03316872), TARE with pembrolizumab (NCT03099564), or with nivolumab (NCT03033446).

## 5. Discussion and Conclusions

Currently approved treatments for advanced HCC still have limited efficacy. In recent years immunotherapy in HCC has been of great interest, especially among ICIs targeting PD-1/PD-L1 and CTLA-4. Following the promising outcomes revealed from phase II trials, phase III trials have been conducted with nivolumab against sorafenib for patients naive to systemic therapy and with pembrolizumab against best supportive care for the treatment of sorafenib-exposed patients. However, in these phase III trials the primary endpoints of OS improvement with nivolumab or pembrolizumab were not statistically significant. More recently, results from the primary analysis of the phase III trial evaluating atezolizumab plus bevacizumab compared to sorafenib have reported a significant survival benefit in patients with HCC who had not previously received systemic treatments. Considering that a substantial proportion of patients with HCC fail to derive clinical benefit from immune checkpoint blockade, and as yet, few predictive biomarkers have been found to select patients with HCC who may benefit, ICIs remain to be further investigated in HCC treatment, as well as whether ICIs in virus-related HCC can control virus relapse. Several strategies aiming at increasing ICIs efficacy and improving patient selection for the treatment are currently being explored. Understanding resistance to ICIs is important in order to improve their outcome by means of combination therapies. Mechanisms of resistance can involve tumor immunogenicity, antigen presentation, generation of tumor-specific T cells, effective tumor cell killing. Multiple studies are evaluating ICI-based combinatorial therapies, in particular with anti-angiogenics, loco-regional treatments or even a dual PD1 and CTLA-4 blockade. Interestingly, recent phase III IMbrave150 trial [84] has shown promising results for the use of ICIs plus anti-angiogenic agent, thus making increasingly clear that a combination strategy with a well-defined scientific rationale is necessary. Moreover, since HCC tumors are enriched with Tregs [32], strategies that inhibit Treg in combination with ICIs, should deserve attention and it is reasonable that we will have to adopt the use of triplets in the near future. With respect to patient selection, subgroup analysis of clinical trials could provide precious information on potential predictive factors. In the case of tremelimumab, patients with objective responses were found to have higher TILs infiltration after treatment compared with non-responders [98]. Conversely, baseline expression of PD-L1 on tumor cell did not show to have an impact on the objective response rates to nivolumab [87]. In fact, tumor biopsies collected at the baseline within the CheckMate 040 trial, were retrospectively assessed for PD-L1 status. Response to therapy was observed in 26% patients with PD-L1 expression and in 19% patients without PD-L1 expression, with no significant difference between the two groups. In addition, a small phase II study of pembrolizumab in HCC did not show a correlation between response and PD-L1 tumor staining [99]. Whether other serum or tissue biomarkers could be useful is still an open question, particularly for PD-L1 expression in stromal cells as well as infiltrating-cell subsets. Indeed, knowledge is improving on the intricate patterns of T-cell populations inside HCC tumors. Recently, a study aiming at characterizing molecular features of immune cells infiltrating HCCs showed high expression levels of PD-1/PD-L1 as well as of markers of inflammatory response [43]. These findings suggest that a subgroup of patients with HCC might be highly responsive to ICIs or other immunotherapeutic agents, although prospective clinical trials are needed to confirm this hypothesis. Importantly, HCC-mediated immune tolerance in the unique tolerogenic environment of the liver itself should be taken into account for developing successful treatments and thus designing clinical trials optimally for HCC. A deeper understanding of the mechanisms underlying HCC immunology will allow research to direct toward a better design of treatments that counteract both the innate and adaptive immune response. In conclusion, clinical trials evaluating mono- or combinatorial immunotherapies including ICIs in HCC are underway, as well as studies on the mechanisms underlying combination strategies and the identification of predictive biomarkers of response and immune-related adverse events, in order to increase clinical benefit and avoid inappropriate treatments, thus minimizing potential enhanced toxicities, given the coexisting liver dysfunction in patients with HCC. Results from these studies will allow to treat patients with HCC in a safer and more effective way implementing a more personalized immunotherapy. The results of the IMbrave 150 study could be really transformative for patients with advanced HCC since for the first time a regimen has markedly improved survival over sorafenib. From the IMbrave150 trial, it can be expected that the combination of atezolizumab plus bevacizumab will represent the new standard for untreated patients in the near future. Furthermore, it has paved the way to further explore immunotherapy-based combination strategies having a well-defined scientific rationale.

## Figures and Tables

**Table 1:** Summary of clinical trials conducted with anti-PD-1, anti-PDL-1, and anti-CTLA-4 monotherapy in HCC.

Drug	Trial	Phase	Sample Size	Treatment Setting	ORR%	DCR%	PFS Months	OS Months
Nivolumab	Checkmate-040	I/II	214 *	First-line ff	20	64	4	NR
	Checkmate-459	III **	743	First-line	15 vs. 7	NA	3.7 vs. 3.8	16.4 vs. 14.7
Pembrolizumab	KEYNOTE-224	II	104	Second-line	17	61	4.9	12.9
	KEYNOTE-240	III ***	413	Second-line	18.3 vs. 4.4	62.2 vs. 53.3	3 vs. 2.8	13.9 vs. 10.6
Tislelizumab	NCT02407990	I	45	Pre-treated	12.2	51	NA	NA
Durvalumab	NCT01693562	I/II	39	Pre-treated	10.3	33.3	NA	13.2
Atezolizumab	GO30140	Ib ****	NA	First-line	NA	NA	3.4 vs. 5.6	NA
Tremelimumab	NCT01008358	II	20	Second-line ff	17.6	76.4	6.48	8.2

ff.: and the followings; NR: not reached; NA: not available. * expansion phase; ** vs. sorafenib; *** vs. placebo; **** vs. atezolizumab plus bevacizumab.

**Table 2:** Summary of ongoing clinical trials with anti-PD-1 and anti-PDL-1 monotherapy in HCC.

Drug	Trial	Phase	Treatment Setting	Estimated Sample Size	Primary Endpoint
Nivolumab	CheckMate-9DX	III (vs placebo)	Adjuvant	530	RFS *
Pembrolizumab	KEYNOTE-394	III (vs placebo)	Second-line (Asian pts **)	450	OS
	KEYNOTE-937	III (vs placebo)	Adjuvant	950	RFS*
Tislelizumab	RATIONALE 301	III (vs sorafenib)	First-line	674	OS
Avelumab	NCT03389126	II	Second-line	30	RR ***

* Recurrence-free survival; ** patients; *** response rate.

## Abbreviations

CTLA-4	Cytotoxic T-Lymphocyte Antigen 4
DCR	Disease Control Rate
DCs	Dendritic cells
FDA	Food and Drug Administration
HCC	Hepatocellular carcinoma
ICIs	Immune checkpoint inhibitors
Ig	Immunoglobulin
MKIs	Multi-kinase inhibitors
ORR	Overall Response Rate
OS	Overall Survival
PD-1	Programmed Death 1
PD-L1	PD-1/Programmed Death-ligand
PFS	Progression-free Survival
TACE	Trans-arterial chemoembolization
TARE	Trans-arterial radioembolization
VEGFR	Vascular Endothelial Growth Factor
